# CDDO-Me Attenuates Vasogenic Edema and Astroglial Death by Regulating NF-κB p65 Phosphorylations and Nrf2 Expression Following Status Epilepticus

**DOI:** 10.3390/ijms20194862

**Published:** 2019-09-30

**Authors:** Min-Ju Kim, Hana Park, Seo-Hyeon Choi, Min-Jeong Kong, Ji-Eun Kim, Tae-Cheon Kang

**Affiliations:** 1 Department of Anatomy and Neurobiology, College of Medicine, Hallym University, Chuncheon 24252, Korea; M19050@hallym.ac.kr (H.P.); 20161239@hallym.ac.kr (S.-H.C.); kmj4180@hallym.ac.kr (M.-J.K.); jieunkim@hallym.ac.kr (J.-E.K.); 2 Institute of Epilepsy Research, College of Medicine, Hallym University, Chuncheon 24252, Korea

**Keywords:** AKT, astrocyte, BBB, eNOS, microglia, PI3K, seizure, SMI-71

## Abstract

2-Cyano-3,12-dioxo-oleana-1,9(11)-dien-28-oic acid methyl ester (CDDO-Me) is a triterpenoid analogue of oleanolic acid that has anti-inflammatory, antioxidant, and neuroprotective activities. In the present study, we evaluate the effects of CDDO-Me on serum extravasation and astroglial death in the rat piriform cortex (PC) induced by status epilepticus (a prolonged seizure activity, SE) in order to propose an underlying pharmacological mechanism of CDDO-Me and its availability for treatment of vasogenic edema. CDDO-Me effectively mitigated serum extravasation and a massive astroglial loss in the PC following SE. CDDO-Me abrogated tumor necrosis factor-α (TNF-α) synthesis in activated microglia by inhibiting nuclear factor-κB (NF-κB) p65 serine 276 phosphorylation. CDDO-Me also abolished NF-κB threonine 435 phosphorylation in endothelial cells and TNF-α-mediated-phosphatidylinositol-3-kinase (PI3K)/AKT/endothelial nitric oxide synthase (eNOS) signaling cascades, which trigger vasogenic edema following SE. Furthermore, CDDO-Me increased astroglial viability via the up-regulation of nuclear factor-erythroid 2-related factor 2 (Nrf2) expression. Therefore, our findings suggest that CDDO-Me may ameliorate SE-induced vasogenic edema formation by regulating NF-κB p65 phosphorylations in microglia as well as endothelial cells and enhancing Nrf2 expression in astrocytes, respectively.

## 1. Introduction

Vasogenic edema results from the increased capillary permeability due to breakdown in intact brain–blood barrier (BBB) that is important for the maintenance of brain homeostasis [1,2]. Serum extravasation during vasogenic edema formation leads to spreading depolarizations and epileptiform discharges [3]. In addition, the leakage of albumin from blood into brain tissue activates microglia and results in the production of inflammatory mediators [4,5], although astrocytes and blood-derived monocytes are also involved in pro-inflammatory reactions [6,7]. These neuroinflammatory responses to vasogenic edema formation are the risk factors of pharmacoresistant temporal lobe epilepsy that is uncontrolled by conventional antiepileptic drugs [8]. This is because multidrug efflux transporter expressions are up-regulated during the recovery of vasogenic edema [9]. Therefore, the blockade or attenuation of vasogenic edema formation may be one of the important therapeutic strategies for the prevention of secondary complications following various brain insults including status epilepticus (SE, a prolonged seizure activity).

The underlying mechanisms of BBB disruption involve various signaling pathways, such as phosphatidylinositol-3-kinase (PI3K), AKT [10], matrix metalloproteinase-9 [2], and endothelin-1 [11]. In particular, tumor necrosis factor-α (TNF-α)-mediated nuclear factor-κB (NF-κB) p65-threonine (T) 435 phosphorylation initiates up-regulations of endothelin B (ET_B_) receptor and transient receptor potential canonical channel-3 (TRPC3), which increase endothelial nitric oxide synthase (eNOS) expression via PI3K/AKT signaling pathway following SE [1,12,13]. Therefore, TNF-α-induced NF-κB activation is one of the common up-stream regulators of vasogenic edema formation induced by SE.

On the other hand, 2-cyano-3,12-dioxooleana-1,9-dien-28-oic acid methyl ester (CDDO-Me; RTA 402) is a triterpenoid analogue of oleanolic acid that is structurally similar to steroids and has anti-inflammatory properties [14]. CDDO-Me suppresses microglial proliferation and its activation, while it exerts microglial phagocytic activity. CDDO-Me also directly inhibits NF-κB signaling and the transcription of pro-inflammatory genes such as NOS and TNF-α [15,16,17,18,19,20]. Furthermore, CDDO-Me ameliorates warfarin-mediated intracranial hemorrhage by nuclear factor-erythroid 2-related factor 2 (Nrf2) activation [21]. With respect to these previous studies, it is noteworthy to explore the effects of CDDO-Me on SE-induced vasogenic edema formation and its underlying mechanisms, which have been elusive.

Here, we demonstrate that CDDO-Me effectively mitigated vasogenic edema formation and a massive astroglial loss in the piriform cortex (PC) following SE. CDDO-Me ameliorated microglial activation and TNF-α synthesis by inhibiting NF-κB serine (S) 276 phosphorylation. In addition, CDDO-Me abrogated the NF-κB-T435/PI3K/AKT/eNOS signaling cascade in endothelial cells, and increased Nrf2 expression in astrocytes following SE. However, CDDO-Me did not prevent increase in vascular endothelial growth factor (VEGF) expression following SE. Therefore, these findings suggest that CDDO-Me may attenuate SE-induced vasogenic edema by inhibiting NF-κB p65 phosphorylations in microglia and endothelial cells, and enhancing Nrf2 expression in astrocytes, respectively.

## 2. Results

### 2.1. CDDO-Me Effectively Attenuates SE-Induced Vasogenic Edema in the PC

**Figure 1:**
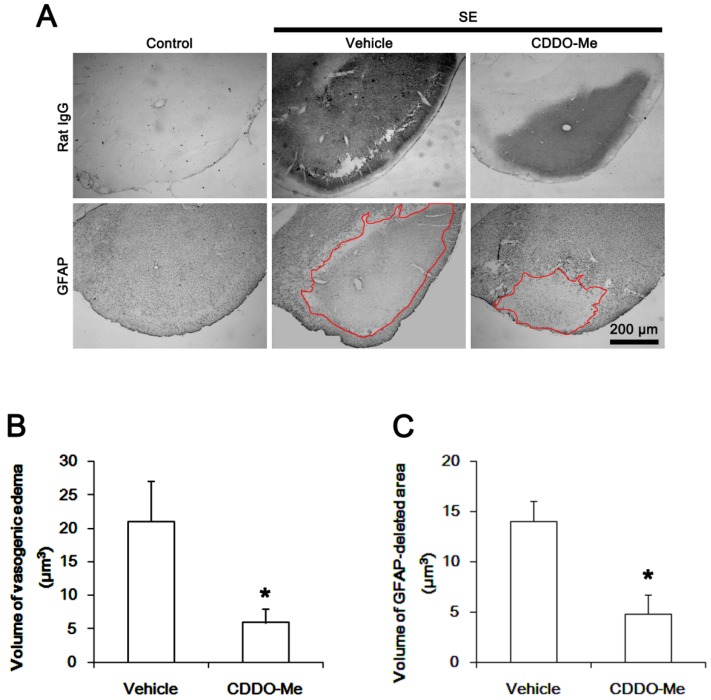
Effects of CDDO-Me on vasogenic edema formation and astroglial loss in the piriform cortex (PC) following status epilepticus (SE). CDDO-Me attenuates serum extravasation and astroglial degeneration induced by SE. (**A**) Representative photographs for vasogenic edema and astroglial loss in the PC. Red lines indicate the contours of vasogenic edema and glial fibrillary acidic protein (GFAP)-deleted lesion. (**B**,**C**) Quantitative values (mean ± S.E.M) of the effect of CDDO-Me on serum extravasation (**B**) and astroglial loss (**C**) in the PC following SE (*n* = 7, respectively). Significant differences are * *p* < 0.05 vs. vehicle-treated animals (Student’s t-test).

The PC is one of the most susceptible brain regions to pilocarpine-induced SE. In this region, vasogenic edema and astroglial loss peaked at 3 days after SE [22,23]. Thus, the PC is a suitable site to evaluate the effects of CDDO-Me on vasogenic edema formation and the related events following SE. Consistent with our previous studies [12,22], the present data showed that SE led to vasogenic edema and a massive astroglial loss in the PC. As compared to vehicle, CDDO-ME showed ~72% and ~66% reductions in vasogenic edema and glial fibrillary acidic protein (GFAP)-deleted lesion, respectively (*p* < 0.05 vs. vehicle, respectively; Student’s t-test, *n* = 7, respectively; Figure 1). Since CDDO-Me does not affect seizure susceptibility in response to pilocarpine [24], these findings indicate that CDDO-Me may effectively ameliorate SE-induced vasogenic edema formation and astroglial loss, independent of seizure activity.

### 2.2. CDDO-Me Inhibits Microglial Activation and TNF-α Synthesis by Abrogating NF-κB S276 Phosphorylation Following SE

**Figure 2:**
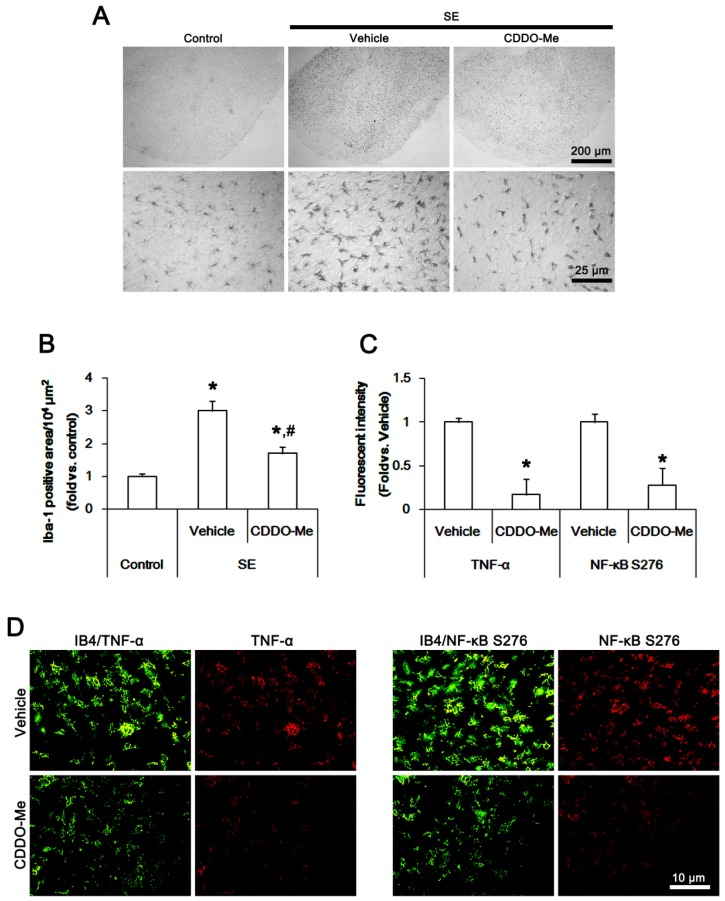
The effect of CDDO-Me on microglia activation in the piriform cortex (PC) following status epilepticus (SE). Ionizing calcium-binding adaptor molecule 1 (Iba-1) positive microglia show hypertrophic morphologies with hyper-ramified processes that are covered by a lot of thorny spines following SE. In addition, SE increases tumor necrosis factor-α (TNF-α) expression and nuclear factor-κB (NF-κB) serine (S) 276 phosphorylation. CDDO-Me abolishes Iba-1 positive microglia transformation, TNF-α expression, and NF-κB S276 phosphorylation induced by SE. (**A**) Representative images for Iba-1 positive microglia. (**B**,**C**) Quantification of the effect of CDDO-Me on Iab-1 positive area (**B**) and the fluorescent intensities of TNF-α and NF-κB S276 signals in microglia (**C**) following SE (*n* = 7, respectively). Significant differences are *,^#^ *p* < 0.05 control- and vehicle-treated animals in panel B, respectively (one-way ANOVA followed by Bonferroni test for multiple comparisons), and *,^#^ *p* < 0.05 vehicle-treated animals in panel C (Student’s *t*-test). (**D**) Representative images for TNF-α expression and NF-κB S276 phosphorylation in microglia following SE.

Next, we evaluated the effect of CDDO-Me on microglial activation induced by SE. In control animals, ionizing calcium-binding adaptor molecule 1 (Iba-1) positive microglia showed a slender ramified and stellate appearance (Figure 2A), which is indicative of resting microglia [25,26,27]. Following SE, Iba-1 positive microglia had hypertrophic, irregularly shaped soma and blunted processes with thorny spines (Figure 2A), indicating activated microglia [25,26,27]. In addition, the Iba-1 positive area was increased to ~3-fold of the control level (*p* < 0.05 vs. control animals, one-way analysis of variance (ANOVA) followed by Bonferroni test for multiple comparisons, *n* = 7, respectively; Figure 2A,B). CDDO-Me inhibited the microglia transformation (Figure 2A) and decreased the Iba-1 positive area to ~1.7-fold of the control level (*p* < 0.05 vs. vehicle, *n* = 7, respectively; Figure 2A,B).

Since the up-regulation of TNF-α expression in activated microglia plays an important role in SE-induced vasogenic edema formation [1,12,13,23], we investigated whether CDDO-Me affects microglial TNF-α expression induced by SE. In control animals, TNF-α positive microglia were rarely observed in the PC. Following SE, TNF-α expression was significantly up-regulated in activated isolectin B4 (IB4) positive microglia. CDDO-Me abolished SE-induced up-regulation of microglial TNF-α expression (*p* < 0.05 vs. vehicle, Student’s t-test, *n* = 7, respectively; Figure 2C,D).

NF-κB S276 phosphorylation is essential for NFκB subunit-dependent cellular responses [28]. NF-κB S276 phosphorylation enhances its transactivation potential and interaction with cAMP response element-binding (CREB) protein, which is important for the microglial activation [29]. In addition, NF-κB S276 phosphorylation exerts TNF-α synthesis [30]. Thus, we also investigated whether CDDO-Me regulates microglial TNF-α expression by inhibiting NF-κB S276 phosphorylation. In control animals, NF-κB S276 phosphorylation was rarely detected in microglia. Following SE, activated IB4 positive microglia showed NF-κB S276 phosphorylation, which was abrogated by CDDO-Me (*p* < 0.05 vs. vehicle, Student’s *t*-test, *n* = 7, respectively; Figure 2C,D). These findings indicate that CDDO-Me may attenuate microglia activation (transformation) and microglial TNF-α synthesis by inhibiting NFκB S276 phosphorylation.

### 2.3. CDDO-Me Decreases Endothelial NF-κB T435 Phosphorylation Following SE

**Figure 3:**
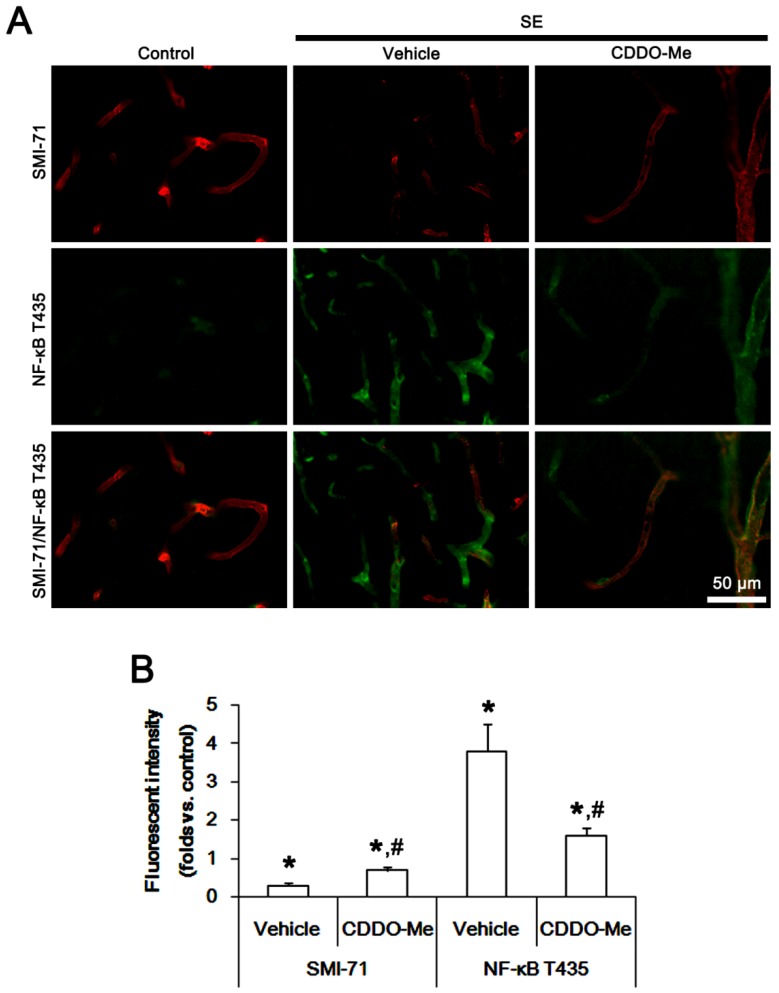
Effects of CDDO-Me on SMI-71 expression and nuclear factor-κB (NF-κB) threonine (T) 435 phosphorylation in the piriform cortex (PC) following status epilepticus (SE). SE diminishes SMI-71 expression in the PC accompanied by the enhanced NF-κB T435 phosphorylation, which are ameliorated by CDDO-Me. (**A**) Representative images for SMI-71 expression and NF-κB T435 phosphorylation. (**B**) Quantification of the effect of CDDO-Me on fluorescent intensities of SMI-71 and NF-κB T435 signals following SE (*n* = 7, respectively). Significant differences are *,^#^ *p* < 0.05 control- and vehicle-treated animals (one-way ANOVA followed by Bonferroni test for multiple comparisons).

We have reported that TNF-α-mediated NF-κB T435 phosphorylation in endothelial cells increases BBB permeability following SE [1]. Therefore, it is likely that CDDO-Me may also attenuate vasogenic edema via the regulation of NF-κB T435 phosphorylation in endothelial cells. To confirm this possibility, we explored its effect on endothelial NF-κB T435 phosphorylation in the PC. As compared to control animals, NF-κB T435 phosphorylation was increased in endothelial cells following SE, accompanied by the reduced SMI-71 (an endothelial barrier antigen) expression (*p* < 0.05 vs. control animals, one-way ANOVA followed by Bonferroni test from multiple comparisons, *n* = 7; Figure 3A,B). CDDO-Me effectively alleviated the enhanced NF-κB T435 phosphorylation and the reduced SMI-71 expression in endothelial cells induced by SE (*p* < 0.05 vs. vehicle; Figure 3A,B). These findings indicate that CDDO-Me may also ameliorate SE-induced vasogenic edema formation via blockade of TNF-α-mediated NF-κB T435 activation in endothelial cells.

### 2.4. CDDO-Me Inhibits PI3K/AKT/eNOS Signaling Pathway Following SE

**Figure 4:**
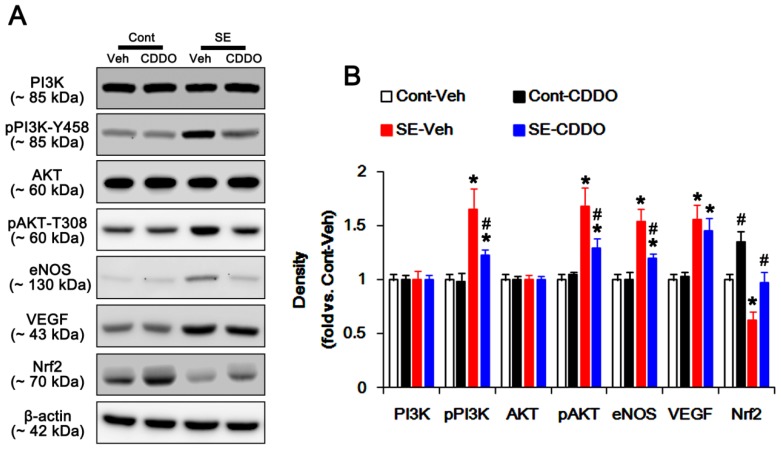
Effects of CDDO-Me on expressions and phosphorylations of phosphatidylinositol-3-kinase (PI3K), AKT, endothelial nitric oxide synthase (eNOS), vascular endothelial growth factor (VEGF), and nuclear factor-erythroid 2-related factor 2 (Nrf2) in the piriform cortex (PC) following status epilepticus (SE). SE increases phosphorylations of PI3K and AKT as well as expressions of eNOS and VEGF. CDDO-Me abrogates the up-regulations of PI3K/AKT phosphorylations and eNOS expressions induced by SE, without altering VEGF expression. In addition, CDDO-Me increases Nrf2 expression level in the PC of control animals. SE decreases Nrf2 expression, which is abolished by CDDO-Me. (**A**) Western blot image for expressions and phosphorylations of PI3K, AKT, eNOS, VEGF, and Nrf2 following SE. (**B**) Quantification of the effect of CDDO-Me on expressions and phosphorylations of PI3K, AKT, eNOS, VEGF, and Nrf2 (*n* = 7, respectively). Significant differences are *,^#^ *p* < 0.05 control- and vehicle-treated animals (one-way ANOVA followed by Bonferroni test for multiple comparisons).

Since NF-κB activation triggers the PI3K/AKT/eNOS signaling pathway during vasogenic edema formation [10,13], we investigated if CDDO-Me inhibits PI3K/AKT phosphorylation and eNOS expression following SE. Under physiological condition, CDDO-Me did not affect PI3K/AKT phosphorylations and eNOS expressions in the PC (Figure 4A,B). SE significantly increased pPI3K-tyrosine (Y) 458 and pAKT-T308 phosphorylations to 1.65- and 1.68-fold of the control level in the PC (*p* < 0.05 vs. control animals, one-way ANOVA followed by Bonferroni test for multiple comparisons, *n* = 7, respectively; Figure 4A,B). SE also elevated expressions of eNOS and VEGF to 1.54- and 1.56-fold of the control level in the PC (*p* < 0.05 vs. control animals, one-way ANOVA followed by Bonferroni test for multiple comparisons, *n* = 7, respectively; Figure 4A,B). CDDO-Me effectively prevented the up-regulation of PI3K/AKT phosphorylations and eNOS expression to 1.23-, 1.29-, and 1.2-fold of the control level following SE (*p* < 0.05 vs. control animals, one-way ANOVA followed by Bonferroni test for multiple comparisons, *n* = 7, respectively; Figure 4A,B). However, CDDO-Me did not influence the increased VEGF expression following SE (Figure 4A,B). Since CDDO-Me could not influence PI3K/AKT phosphorylations and eNOS expression under physiological conditions, our findings support the possibility that CDDO-Me may inhibit the PI3K/AKT/eNOS signaling pathway via blockade of TNF-α synthesis or TNF-α-mediated NF-κB p65 phosphorylations induced by SE.

### 2.5. CDDO-Me Mitigates SE-Induced Astroglial Loss by Enhancing Nrf2 Expression

SE results in acute and devastating astroglial degeneration in the PC, which is characterized by a pattern of selective vulnerability [22,31,32,33]. Astroglial loss/dysfunctions also aggravate vasogenic edema following SE [12,22]. In the present study, CDDO-Me ameliorated SE-induced astroglial loss in the PC, concomitant with the reduced vasogenic edema formation (Figure 1A–C). Thus, the remaining question is how CDDO-Me would protect astroglial damage from SE. Interestingly, CDDO-Me is an activator of Nrf2 that is a master mediator of the cellular antioxidant response. Furthermore, CDDO-Me up-regulates Nrf2 expression in astrocytes [21,34]. Since the production of intracellular reactive oxygen species by NADPH oxidase in astrocytes is involved in SE-induced astroglial death [12], it is likely that CDDO-Me may mitigate astroglial degeneration by increasing Nrf2 expression following SE. To confirm this hypothesis, we explored the effect of CDDO-Me on astroglial Nrf2 expression under physiological- and post-SE conditions. In the present study, Western blots demonstrated that CDDO-Me increased Nrf2 expression level to 1.35-fold of vehicle level in the PC of control animals (*p* < 0.05 vs. vehicle, one-way ANOVA followed by Bonferroni test for multiple comparisons, *n* = 7, respectively; Figure 4A,B). Following SE, Nrf2 expression was decreased to 0.62-fold of control level (*p* < 0.05 vs. vehicle-treated control animals, one-way ANOVA followed by Bonferroni test for multiple comparisons, *n* = 7, respectively; Figure 4A,B). CDDO-Me effectively ameliorated the reduction in SE-induced Nrf2 expression (*p* < 0.05 vs. vehicle, one-way ANOVA followed by Bonferroni test for multiple comparisons, *n* = 7, respectively; Figure 4A,B).

**Figure 5:**
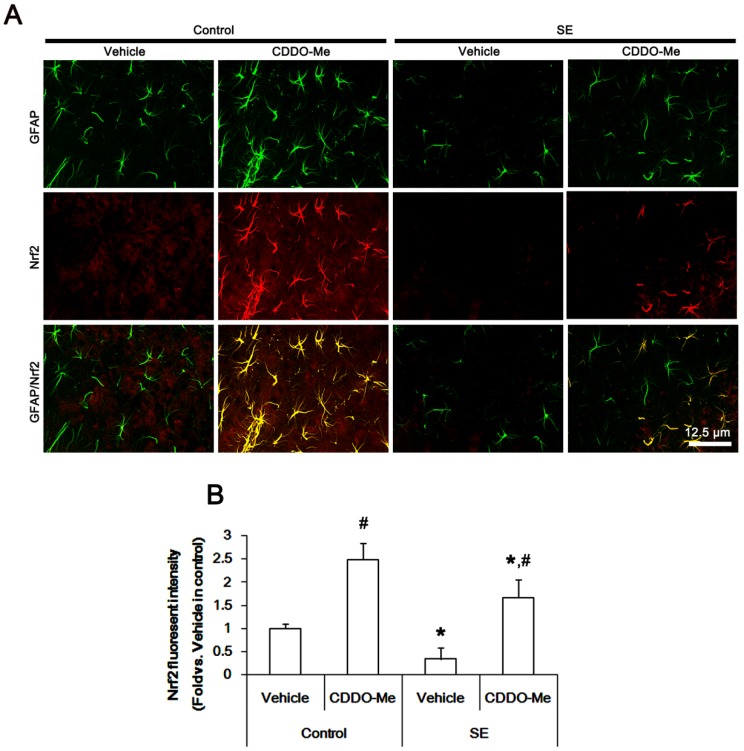
Effects of CDDO-Me on astroglial nuclear factor-erythroid 2-related factor 2 (Nrf2) expression in the piriform cortex (PC) following status epilepticus (SE). Under physiological conditions, CDDO-Me up-regulates Nrf2 expression in astrocytes rather than other cells. Following SE, Nrf2 expression is reduced in both astrocytes and other cells. CDDO-Me mitigates SE-induced reduction in Nrf2 expression in astrocytes rather than other cells. (**A**) Representative images for astroglial Nrf2 expression in the PC following SE. (**B**) Quantification of the effect of CDDO-Me on Nrf2 fluorescent intensity (*n* = 7, respectively). Significant differences are *,^#^ *p* < 0.05 control- and vehicle-treated animals (one-way ANOVA followed by Bonferroni test for multiple comparisons).

Consistent with previous studies [21,34], immunohistochemical studies revealed that Nrf2 expression was observed in astrocytes within the PC of control animals (Figure 5A). CDDO-Me up-regulated Nrf2 expression in astrocytes more than other cell populations under physiological condition (*p* < 0.05 vs. vehicle-treated control animals, one-way ANOVA followed by Bonferroni test for multiple comparisons, *n* = 7, respectively; Figure 5A,B). Following SE, Nrf2 expression was reduced in all cell populations. Remaining (surviving) astrocytes showed Nrf2 expression (*p* < 0.05 vs. vehicle-treated control animals, one-way ANOVA followed by Bonferroni test for multiple comparisons, *n* = 7, respectively; Figure 5A,B). CDDO-Me ameliorated SE-induced reduction in Nrf2 expression in astrocytes more than other cell populations (*p* < 0.05 vs. vehicle, one-way ANOVA followed by Bonferroni test for multiple comparisons, *n* = 7, respectively; Figure 5A,B). These findings suggest that CDDO-Me may attenuate SE-induced astroglial degeneration by enhancing Nrf2 expression and/or preventing vasogenic edema formation.

## 3. Discussion

Vasogenic edema is the most common type of brain edema due to BBB disruption, which results in an abrupt increase in intracranial pressure, abnormal blood–brain transports of serum-derived molecules, and aberrant neuronal excitability [1,2,3]. Although the underlying mechanisms of vasogenic edema formation are very complicated, neuroinflammatory responses to harmful stimuli are emphasized. In particular, TNF-α is thought to be one of the up-stream regulators for vasogenic edema formation induced by SE, since blockade of TNF-α functions by soluble TNF p55 receptor ameliorates vasogenic edema through abrogating activations of NF-κB, eNOS, PI3K, and AKT [1,12,13]. Consistent with these previous studies, the present study shows that TNF-α expression was rapidly upregulated in activated microglia following SE. CDDO-Me attenuated vasogenic edema formation by inhibiting microglial TNF-α expression induced by SE. CDDO-Me also abolished activations/up-regulations of the down-stream effectors in TNF-α-mediated signaling pathway, such as PI3K, AKT, and eNOS, following SE. However, CDDO-Me did not influence the up-regulated VEGF expression following SE. Considering the inhibitory effect of CDDO-Me on TNF-α production [15,16], our findings indicate that CDDO-Me may attenuate SE-induced vasogenic edema formation by affecting the TNF-α-mediated PI3K/AKT/eNOS signaling pathway, independent of the up-regulated VEGF expression.

In the present study, CDDO-Me inhibited NF-κB S276 and T435 phosphorylations in microglia and endothelial cells, respectively, following SE. Similar to microglial TNF-α synthesis, SE also up-regulates TNF receptor expressions in astrocytes (TNFp55 and TNFp75 receptors) and endothelial cells (TNFp75 receptor), which are relevant to vasogenic edema formation [1,12]. TNF receptor activations increase NF-κB phosphorylation that enhances its transactivation potential [29,35]. NF-κB S276 phosphorylation plays an important roles in microglial activation and TNF-α synthesis [23,29,30]. Furthermore, NF-κB T435 phosphorylation in endothelial cells results in vasogenic edema induction via SMI-71 degradation [1]. Since CDDO-Me directly inhibits NF-κB signaling [16,17], our findings suggest that the inhibition of NF-κB S276 phosphorylation by CDDO-Me may abrogate microglial TNF-α production and the subsequent endothelial NF-κB T435 phosphorylation, which would mitigate vasogenic edema formation induced by SE.

Astrocytes are the most numerous non-neuronal cell types in the brain, which participate in the BBB integrity. Thus, the dysfunctions of astrocytes as well as endothelial cells induce BBB breakdown leading to vasogenic edema [12,22]. Recent studies have revealed that astroglial subpopulations show differential vulnerability in regional-specific patterns following SE, independent of hemodynamics [36]. In particular, the PC is the most susceptible brain region to SE-induced astroglial degeneration [12,22,36]. Consistent with these previous reports, the present data show that SE resulted in a massive astroglial loss in the PC, accompanied by reduced Nrf2 expression. Nrf2 is a redox-sensitive transcription factor, which maintains redox homeostasis by regulating antioxidant-response element (ARE)-dependent transcription and antioxidant defense enzymes [37,38]. Under physiological condition, Kelch-like ECH-associated protein 1 (Keap1) binds to Nrf2, which inhibits nuclear Nrf2 translocation and facilitates Nrf2 degradation via the ubiquitin–proteasome system [34,38,39,40]. Thus, up-regulation of endogenous Nrf2 expression is not sufficient to prevent cell injuries under pathophysiological conditions [21,34]. Since NADPH oxidase-mediated free radical production triggers SE-induced astroglial death in the PC [12], our findings indicate that the decreased Nrf2 expression may be relevant to a massive astroglial loss in the PC. Indeed, the present data demonstrate that CDDO-Me attenuated SE-induced vasogenic edema and astroglial loss, accompanied by up-regulation of Nrf2 expression. Furthermore, CDDO-Me increased Nrf2 expression in astrocytes under physiological conditions. CDDO-Me dissociates Keap1 from Nrf2 by interacting with the reactive cysteine 151 residue of Keap1 through a Michael addition [41], which abrogates Keap1-mediated Nrf2 ubiquitination and results in Nrf2 accumulation/activation [42]. In addition, CDDO-Me itself exerts Nrf2 transcription [43,44]. Therefore, it is likely that CDDO-Me may also inhibit vasogenic edema formation by increasing astroglial viability. However, CDDO-Me increased Nrf2 expression in astrocytes more than neurons under physiological- and post-SE conditions. Since Nrf2 activation protects neurons from ischemia via astrocytes [34], these findings suggest that CDDO-Me may affect Nrf2 expression in astrocytes rather than neurons. Further studies are needed to elucidate these astrocyte-friendly CDDO-Me properties.

Although CDDO-Me improves kidney function in patients with chronic kidney disease stage 4 and type 2 diabetes, a phase 3 clinical trial evaluating CDDO-Me was terminated for safety concerns. This is because CDDO-ME increased the risk of heart failure hospitalizations or death from heart failure [45]. Therefore, the development of a novel BBB-permeable derivative of CDDO-Me would be needed for clinical trials concerning prevention treatment for vasogenic edema.

## 4. Materials and Methods

### 4.1. Experimental Animals and Chemicals

The present study was carried out on adult male Sprague–Dawley (SD) rats (7 weeks old). Animals were housed in a controlled room temperature (22 ± 2 °C), humidity (55 ± 5%), and a light–dark cycle on a 12-h on-off cycle. Food and water were available ad libitum throughout the experiments. All experimental protocols described below were approved by the Institutional Animal Care and Use Committee of Hallym University (Chuncheon, South Korea, Hallym 2018-2, 26th, April 2018). Every effort was made to reduce the number of animals employed and to minimize animal discomfort. All reagents were obtained from Sigma-Aldrich (St. Louis, MO, USA), except as noted.

### 4.2. Surgery and Drug Infusion

Under Isoflurane anesthesia (3% induction, 1.5–2% for surgery, and 1.5% maintenance in a 65:35 mixture of N_2_O:O_2_), animals were infused with vehicle or CDDO-Me into the right lateral ventricle (1 mm posterior; 1.5 mm lateral; −3.5 mm depth to the bregma) with a brain infusion kit 1 and an Alzet 1007D osmotic pump (Alzet, Cupertino, CA, USA). The osmotic pump contained vehicle or CDDO-Me (10 μM). The pump was placed in a subcutaneous pocket in the dorsal region. In a pilot study and our previous study [24], this dosage of CDDO-Me did not show behavioral and neurological defects and could not change the seizure susceptibility and seizure severity in response to pilocarpine. Three days after surgery, rats were induced with SE by lithium chloride (LiCl)-pilocarpine.

### 4.3. SE Induction

SE was induced by a single dose (30 mg/kg) of pilocarpine in rats pretreated (24 h before pilocarpine injection) with 127 mg/kg LiCl. Before pilocarpine injection, animals were given atropine methylbromide (5 mg/kg i.p.) to block the peripheral effects of pilocarpine. As controls, rats were treated with saline instead of pilocarpine. Two hours after SE onset, diazepam (Valium; Roche, Neuilly sur-Seine, France; 10 mg/kg, i.p.) was administered to terminate SE and repeated, as needed [22,23,24,25,26]. Three days after SE, animals were used for immunohistochemistry and Western blot.

### 4.4. Tissue Processing

Under urethane anesthesia (1.5 g/kg, i.p.), animals were perfused via a cannula into the left ventricle of the heart with 0.9% saline followed by 4% paraformaldehyde in 0.1 M phosphate buffer (PB, pH 7.4). After perfusion, the brains were removed and post-fixed in the same fixative overnight, and subsequently cryoprotection was conducted with 30% sucrose/0.1 M PBS. Brain coronal sections of 30 μm were obtained with a cryo-microtome. For Western blot, animals were decapitated under urethane anesthesia. The PC was rapidly dissected out and homogenized in lysis buffer. The protein concentration in the supernatant was determined using a Micro BCA Protein Assay Kit (Pierce Chemical, Dallas, TX, USA).

### 4.5. Immunohistochemistry

Free-floating sections were washed 3 times in PBS (0.1 M, pH 7.3). Next, to inactivate the endogenous peroxidase, sections were incubated in 3% H_2_O_2_ and 10% methanol in PBS (0.1 M) for 20 min at room temperature. Later, sections were incubated in primary antibody (Table 1). Tissue sections were developed in 3,3′-diaminobenzidine in 0.1 M Tris buffer and mounted on gelatin-coated slides. Some sections were incubated with a cocktail solution containing the primary antibodies or IB4 (Table 1) in PBS containing 0.3% Triton X-100 overnight at room temperature. Thereafter, sections were visualized with appropriate Cy2- and Cy3-conjugated secondary antibodies. Immunoreaction was observed using an Axio Scope microscope (Carl Zeiss Korea, Seoul, South Korea). To establish the specificity of the immunostaining, a negative control test was carried out with preimmune serum instead of the primary antibody. No immunoreactivity was observed for the negative control in any structures. All experimental procedures in this study were performed under the same conditions and in parallel.

### 4.6. Measurements of Volumes of Vasogenic Edema and GFAP-Deleted Lesion, Iba-1 Positive Area, and Fluorescent Intensities

The volumes of vasogenic edema and GFAP-deleted lesion were measured by the modified Cavalieri method. Areas of vasogenic edema and GFAP-deleted lesion were measured by AxioVision Rel. 4.8 software (Carl Zeiss Korea, Seoul, South Korea). Thereafter, the volumes (V) were estimated according to the formula: V = Σ*a* × *t*_nom_ × 1/ssf, where *a* is area, *t*_nom_ is the nominal section thickness (of 30 μm in this study), and ssf is the section sampling fraction (of 1/6 in this study) [20]. Iba-1 positive area was also measured, as previously described [26]. Briefly, sections (10 sections per each animal, *n* = 7 in each group) were captured, and areas of interest (1 × 10^4^ μm^2^) were selected. Thereafter, measurement of Iba-1 positive area was performed on 20× images using AxioVision Rel. 4.8 software. To measure fluorescent intensity, 30 areas/rat (300 μm^2^/area) were randomly selected within the PC (15 sections from each animal, *n* = 7 in each group). Thereafter, mean fluorescence intensities of TNF-α, SMI-71, NFκB T435, and Nrf2 signals on each section were measured by using AxioVision Rel. 4.8 software. Intensity measurements were represented as the number of a 256 gray scale. Intensity of each section was standardized by setting the threshold level (mean background intensity obtained from five image inputs). Manipulation of the images was restricted to threshold and brightness adjustments to the whole image. Measurements of volumes of vasogenic edema and GFAP-deleted lesion, Iba-1 positive area, and fluorescent intensities were performed by two different investigators who were blind to the classification of tissues.

### 4.7. Western Blot

Western blot was performed by the standard protocol (*n* = 7 in each group). The primary antibodies used in the present study are listed in Table 1. The bands were detected and quantified on an ImageQuant LAS4000 system (GE Healthcare Korea, Seoul, South Korea). As an internal reference, rabbit anti-β-actin primary antibody (1:5000) was used. The values of each sample were normalized with the corresponding amount of β-actin. The ratio of phosphoprotein to total protein was described as the phosphorylation level.

### 4.8. Data Analysis

All data obtained from the quantitative measurements were analyzed using Student’s t-test and one-way ANOVA to determine statistical significance. Bonferroni’s test was used for post hoc comparisons. A *p*-value below 0.05 was considered statistically significant.

## 5. Conclusions

To the best of our knowledge, the present data validate, for the first time, the protective effects of CDDO-Me against SE-induced vasogenic edema formation and astroglial loss in the PC. Briefly, CDDO-Me attenuated vasogenic edema by inhibiting NF-κB S276 and T435 phosphorylations in microglia and endothelial cells, which abrogated TNF-α production and BBB disruption induced by SE, respectively. Furthermore, CDDO-Me protected astrocytes from SE via the up-regulation of Nrf2 expression. Therefore, these findings propose the underlying pharmacological mechanisms of CDDO-Me and its derivates against vasogenic edema formation and astroglial degeneration following SE.

## Figures and Tables

**Table 1:** Primary antibodies and lectin used in the present study.

Antigen	Host	Manufacturer (Catalog Number)	Dilution Used
AKT	Rabbit	Cell signaling (#9272)	1:1000 (WB)
eNOS	Rabbit	Abcam (#ab66127)	1:1000 (WB)
GFAP	Mouse	Millipore (#MAB3402)	1:5000 (IH)
IB4		Vector (B-1205)	1:200 (IH)
Iba-1	Rabbit	Biocare Medical (CP 290)	1:500 (IH)
NFκB S276	Rabbit	Abcam (ab106129)	1:100 (IH)
NFκB T435	Rabbit	Abcam (ab31472)	1:100 (IH)
Nrf2	Rabbit	Abcam (ab137550)	1:1000 (WB), 1:100 (IH)
pAKT-T308	Rabbit	Cell signaling (#9275)	1:1000 (WB)
pPI3K-Y458	Rabbit	Cell signaling (#4228S)	1:1000 (WB)
PI3K	Rabbit	Cell signaling (#4292S)	1:1000 (WB)
Rat IgG	Goat	Vector (#PI-9400)	1:200 (IH)
SMI-71	Mouse	Covance (#SMI-71R)	1:1000 (IH)
TNF-α	Goat	R&D systems (AF-510-NA)	1:1000 (IH)
VEGF	Rabbit	Abcam (#ab46154)	1:1000 (WB)
β-actin	Mouse	Sigma (#A5316)	1:5000 (WB)

eNOS: Endothelial nitric oxide synthase; GFAP: Glial fibrillary acidic protein; IB4: Isolectin B4; Iba-1: Ionizing calcium-binding adaptor molecule 1; NF-κB S276: Phospho-nuclear factor-κB p65 serine 276 site; NF-κB T435: Phospho-nuclear factor-κB p65 threonine 435 site; Nrf2: Nuclear factor-erythroid 2-related factor 2; pAKT-T308: Phospho-AKT at threonine 308 site; pPI3K-Y458: Phospho-phosphatidylinositol-3-kinase tyrosine 458 site; Phosphatidylinositol-3-kinase (PI3K); Rat IgG: Rat immunoglobulin; TNF-α: Tumor necrosis factor-α; VEGF: Vascular endothelial growth factor; IH: Immunohistochemistry; WB: Western blot.
